# Exposure to Cyantraniliprole Adversely Impacts Fitness of *Harmonia axyridis*: Acute Toxicity and Sublethal Effects on Development, Fecundity and Antioxidant Responses

**DOI:** 10.3390/insects15100773

**Published:** 2024-10-06

**Authors:** Tianshu Zhang, Yongda Yuan, Haiyuan Teng, Dongsheng Wang, Haotian Gu

**Affiliations:** 1 Shanghai Key Laboratory of Protected Horticultural Technology, Eco-Environmental Protection Research Institute, Shanghai Academy of Agricultural Sciences, Shanghai 201403, China; zhangtianshu2000@163.com (T.Z.); yuanyongda@saas.sh.cn (Y.Y.); tenghaiyuan@126.com (H.T.); zb3@saas.sh.cn (D.W.); 2 Shanghai Engineering Research Centre of Low-Carbon Agriculture (SERCLA), Shanghai 201415, China

**Keywords:** cyantraniliprole, *Harmonia axyridis*, development and fecundity, antioxidant enzymes, sublethal effects

## Abstract

**Simple Summary:**

CNAP is a novel anthranilic diamide insecticide and its toxicological profiles pertaining to ladybird beetles remain largely uncharacterized. Through dipping method and topical application, we comprehensively evaluated acute toxicity and sublethal effects of CNAP against *Harmonia axyridis*, a predacious coccinellid commonly utilized in integrated pest management (IPM). Our outcomes indicated that CNAP was moderately toxic to *H. axyridis* and most noxious to 1st-instar larvae, with the least LC_50_ of 86.11 mg/L. When eggs and 1st instars were treated with LC_30_ CNAP, embryonic, larval and pupal durations all significantly dropped, accompanied by reduced pupal weight and pupation rate. Post sublethal treatments on newly emerged females, reproductive proxies displayed dose-effect responses, with daily spawning and vitellin level substantially diminished and the pre-oviposition period retarded. In addition, sublethal exposure to CNAP profoundly disrupted the antioxidant system of females, as evidenced by an induced hormesis effect at LC_10_ and impaired enzymatic activities at LC_30_ over time. Taken together, this study revealed the prospective ecological risk of CNAP and its adverse implications for *H. axyridis* fitness. As such, the practical compatibility of agrochemicals with biocontrol agents should be well assessed before being embraced into IPM for pest control.

**Abstract:**

Extensive utilization of pesticides and their persistent residues inadvertently pose threats to the effectiveness and fitness of biocontrol agents in agroecosystems. However, these ecological consequences are generally disregarded when executing integrated pest management strategies (IPM). Cyantraniliprole (CNAP) serves as a wide-spectrum diamide insecticide and its sublethal effects have been well characterized on multiple insect pests, whereas its impacts on beneficial natural enemies remain unfathomed. Herein we exposed *Harmonia axyridis*, a predacious generalist, to lethal and sublethal concentrations of CNAP via dipping treatment (egg stage) and topical applications (1st-instar stage + adult stage). The acute toxicity tests revealed that LC_50_ of CNAP were 90.11, 86.11 and 240.50 mg/L against embryos, 1st instar nymphs and female adults, respectively, with safety factors ranging from 1.14 to 5.34, suggesting its medium toxicity for *H. axyridis* and larval stage was the most susceptible. The embryonic, larval and pupal durations of coccinellids ecdysed from CNAP-treated eggs and 1st instars were all elongated under sublethal concentrations, of which LC_30_ triggered more pronounced and significant retardations relative to control. Besides, exposed coccinellids displayed substantially diminished pupal mass and pupation rate, most notably for insects molted from the 1st-instar stage upon CNAP sublethal treatments. With respect to reproductive performance, LC_10_ and LC_30_ of CNAP all significantly suppressed female fecundity, as evidenced by reduced vitellin content, a prolonged pre-oviposition period (POP), mitigated laid eggs and the egg hatching rate. Specifically, there existed positive correlations between vitellin level (Vn) and number of eggs deposited by per female, indicative of CNAP affecting fecundity by regulation of Vn. In addition, the antioxidant system was also profoundly disrupted by CNAP, with compromised POD activity at different concentrations over time and induced hormesis of SOD/CAT activities post LC_10_ exposure. Activities of SOD and TAC were enhanced to exert protective functions during the first 48 h, while defense collapsed at 72 h following LC_30_ treatments that depleted all enzymatic activities. We speculated that fitness trade-offs may occur between reproductive capacity and antioxidant defenses to sustain physiological homeostasis in response to CNAP stress. Collectively, this study evaluated the ecological risk of CNAP and unmasked its adverse implications for overall fitness of *H. axyridis*, which highlighted rational application of agrochemicals to conserve biocontrol agents when implementing IPM strategies for sustainable pest control.

## 1. Introduction

As a pivotal element in modern agriculture, pesticides are originally designed for the protection of crop yield by combating pernicious pests, pathogens and weeds, albeit their indiscriminate and/or massive application may elicit ecological risks for beneficial biota, like population decline and biodiversity loss [1,2], notably in the context of ever-increasing pest resistance and chemical residues in cropping systems. As such, when implementing integrated pest management (IPM) approaches for pest control, agrochemicals should be taken with caution to circumvent hidden deleterious implications for natural enemies [3].

Cyantraniliprole (CNAP) belongs to the second-generation anthranilic diamide insecticides and mechanistically modulates ryanodine receptors (RyRs) of insect sarcoplasmic reticulum, resulting in feeding cessation, muscular paralysis and even individual demise [4]. With this novel and unique mode of action, it displays robust insecticidal activities against a suite of hemipteran, lepidopteran, dipteran and coleopteran insect pests [4,5,6,7,8,9]. Correspondingly, CNAP can be delivered diversely in agronomic scenarios, viz., foliar spray, root irrigation, soil mixture and seed treatment [8,9,10]. Despite low mammalian toxicity, given the high structure similarity and sequence homology of RyRs among arthropods [5], it is conceivable that CNAP may unintentionally impinge on nontarget organisms along with the ecosystem services that they provide.

The multicolored Asian lady beetle, *Harmonia axyridis* (Pallas, 1773) (Coleoptera: Coccinellidae), is a well-established generalist predator endemic to East Asia [11] (Benelli et al., 2015). Due to its cosmopolitan distribution, voracious appetite, polyphagous habit, easy rearing and commercial availability, *H. axyridis* has long been mass-reared and released to prey on aphids, small soft-bodied pests, immature coleopterans and other greenhouse insect pests [12,13,14], rendering it an important biocontrol agent in IPM. In addition, representative of natural enemy insects, it has proven an ideal model for examining susceptivity to pesticides in registration trials [15]. Except for high mortality caused by several pyrethroids, earlier studies have documented the benign and slight toxicity of most insecticides against *H. axyridis* under field and laboratory conditions [11,12,16].

Apart from direct lethal effects of pesticides on predatory and parasitic insects, sublethal impacts involving physiological, behavioral, biochemical and demographic aspects engage burgeoning interest and could have more profound repercussions for biological performance [17]. For example, after exposure to chlorantraniliprole (CAP) under LC_10_ and LC_30_, pre-adult developmental periods and preoviposition period (POP) were lengthened, accompanied by reduced adult longevity and fecundity in *H. axyridis* populations, suggesting growth and reproductive toxicity of anthranilic diamide to this coccinellid [13]. Antioxidant enzymes are ubiquitous in aerobic and aerotolerant organisms and their activity can reflect redox status, being sensitive biomarkers to determine sublethal effects of insecticides [18]. Wu et al. [19] demonstrated that, post LC_50_ exposure of CNAP and CAP, superoxide dismutase (SOD) and catalase (CAT) activities in 3rd-instar larvae of *Helicoverpa armigera* markedly mounted, suggesting cellular oxidative stress upon anthranilic diamide applications. Based on these clues, the question thus arises whether CNAP would also elicit similar sublethal effects and to what extent it is noxious to *H. axyridis*.

Insect development undergoes complex life cycles composed of distinct stages at which physiology, behavior and morphology vary greatly (i.e., motionless egg and less motile pupa versus locomotive larva and adult) [20]. Besides, separated developmental phases may present different susceptibilities and responses to toxicants [12,21]. To comprehensively ascertain the toxicological implications of CNAP in *H. axyridis*, herein we performed dipping poisoning and topical application across life stages. The objectives of this investigation were (i) to determine acute toxicity (LC_50_) and risk level of CNAP against eggs, 1st-instar larvae and newly emerged adult females (NEAF), and (ii) to reveal sublethal effects exerted by CNAP on developmental duration, fecundity and antioxidant systems at LC_10_ and LC_30_.

## 2. Materials and Methods

### 2.1. Insects and Chemicals

A colony of adult coccinellids was field-collected from a vegetable patch in Zhuanghang, Shanghai, China (coordinates 30°53′29″ N, 121°23′16″ E) in June 2021. They were identified as *H. axyridis* based on morphological characterization and DNA barcoding of cytochromec oxidase subunit I (COI) genes, which were sequenced by Saiheng Biotechnology Corporation (Shanghai, China). They were maintained in a climate chamber under controlled conditions (25 ± 1 °C, 65 ± 5% relative humidity and a photoperiod of light 16 h: dark 8 h) without exposure to any chemicals. All assays were carried out under the same physical conditions as the stock strain. We fed them with ad libitum pea aphid (*Acyrthosiphon pisum*) and bred them for circa 50 consecutive generations. *A. pisum* was reared with *Vicia faba* seedlings as host plants. Technical-grade CNAP (94% active ingredient) was procured from FMC Agricultural Sciences Co., Ltd. (Shanghai, China). Analytically pure acetone (Sinopharm Chemical Reagent Co., Ltd., Shanghai, China) was used as solvent.

### 2.2. Acute Toxicity Test and Safety Assessment

To determine LC_50_, LC_30_ and LC_10_ of CNAP against eggs, we adopted the immersion method reported by Wang et al. [8] with slight modifications. In brief, a stock solution (2000 mg/L) was yielded by dissolving 0.2128 g of CNAP into 1 L of acetone, then diluting with 0.1% Triton X-100 aqueous solution to a geometric series of desired concentrations. Egg clusters oviposited by the lab-reared strain (<12 h) were soaked in 23.75, 47.5, 95.0, 190.0 and 380.0 mg/L of CNAP for 10 s, respectively, with chemical residues on egg surfaces removed by blotting on a paper towel. After this, egg masses were individually transferred to petri dishes (5 cm in diameter, 1 cluster/dish) lined with moistened filter paper and sealed by Parafilm. They were allowed to incubate under standard conditions for 10 days, during which time dead ones failed to hatch and those viable were counted as the number of neonates. Since acetone possessed no lethality against tested samples [22], a mixture of water and acetone containing 0.1% Triton X-100 was set as control. A total of ca. 300 eggs were recruited for this test, with 50 for each treatment and control.

In terms of 1st-instar larvae and newly emerged adult females (NEAF) (both hatched and emerged within 12 h), CNAP toxicity was determined by the topical application method [13,23]. Briefly, CNAP was diluted with acetone to gradient concentrations, viz., 23.75, 47.5, 95.0, 190.0 and 380.0 mg/L for nymphs, and 95.0, 190.0, 380.0, 760.0 and 1520.0 mg/L for adults, which were narrowed by range-finding tests previously performed. Healthy insects with uniform size were narcotized by CO_2_ for 30 s in an air-tight container before exposure. Anesthetic samples were administered topically with 1 μL of prepared solution on the abdominal ventral side using a micro-applicator (Burkard, Rickmansworth, UK), with the same volume of acetone as control. To avoid cannibalism upon treatments, specimens were transferred to 12-well cell culture plates, individually reared in an artificial incubator at standard conditions with sufficient *A. pisum* supplied. Dead coccinellids did not react when they were lightly touched with a small soft brush and mortality was recorded at 24 h upon exposure. When mortality of the solvent control group was lower than 10%, the results were considered valid. A total of 90 1st-instar nymphs and 360 NEAF were recruited, with 15 individuals for each concentration and control in the nymph bioassays and 60 per treatment in the adult experiments.

The safety assessment for chemical pesticides was modified from specifications of Zhang et al. [24] and Shan et al. [25]. LC_50_ values of CNAP to coccinellids were determined following the above procedures and its recommended field label rate ranged from 45 mg a.i./L to 75 mg a.i./L [26]. The safety factor was estimated by the below equation and risk levels of pesticides were divided into four categories: ① safety factor > 5 for low risk; ② 5 ≥ safety factor > 0.5 for medium risk; ③ 0.5 ≥ safety factor > 0.05 for high risk; and ④ safety factor ≤ 0.05 for extremely high risk.

$$\mathrm{Safety}\;\mathrm{factor}=\frac{{LC}_{50}(mg\;a.i./L)}{recommended\;field\;rate(mg\;a.i./L)}$$

### 2.3. Fitness Alterations after Sublethal Exposure to CNAP

CNAP was delivered to specimens following the same test procedures as described in Section 2.2, except that LC_10_ and LC_30_ concentrations (see Results Section 3.1) were opted to evaluate the developmental toxicity at immature stages and reproductive toxicity as well as biochemical responses at the adult stage. Acetone aqueous solution plus 0.1 % Triton X-100 and acetone served as controls in Section 2.3.1 and Section 2.3.2, respectively. The coccinellid husbandry followed protocols of Benelli et al. [11] and Nawaz et al. [13].

#### 2.3.1. Immature Development Examination

Survivors that withstood LC_10_ and LC_30_ exposure of CNAP were collected and placed in petri dishes (Ø = 10 cm), with at least 90 eggs and 90 1st instars (n = 30 per concentration and control) amenable to pupal mass and pupation rate examinations. The fresh weight of pupae (2-day-old) was measured using a Sartorius BS 224 analytical balance (Sartorius, Germany) and pupation percentage was estimated based on the below formula. To compare developmental time post treatments, at least 30 eggs and 30 neonates (n = 10 per concentration and control) were randomly selected and durations at preimaginal stages were scored, which were registered as embryonic, larval (from 1st to 4th instar) and pupal periods, respectively. All larvae were individually reared with copious foodstuffs to prevent cannibalism. Water loaded on moistened pieces of cotton was replenished daily throughout experiments.

$$\mathrm{Pupation}\;\mathrm{rate}\;(\%)=\frac{The\;number\;of\;pupated\;4th\;instar\;larve}{The\;total\;number\;of\;4th\;instar\;larve}\;\times 100$$

#### 2.3.2. Reproductive Performance Evaluation

To mimic realistic conditions for a promiscuous species with multiple male partners available simultaneously, each LC_10_ and LC_30_ CNAP-treated NEAF was paired with two wild-type males (0–12 h old), and this grouping was set as one experimental unit and introduced into disposable plastic cups (5 cm diameter, 10 cm height) containing *A. pisum* as a food source and moist cotton as a water source. They were kept in a climatron under standard conditions and left for 48 h to ensure copulations. The male coccinellids were then removed and we continued to monitor female reproductive behavior thereafter. At least 30 units were established, with 10 females recruited per group and one female as one replication. Pieces of folded filter paper were provisioned as oviposition substrate, which was refreshed daily to check for the presence of eggs, and the time elapsed from adult emergence to first spawning was identified as POP. The number of eggs deposited by each female was counted under a stereo microscope Olympus SZ61 (Olympus, Tokyo, Japan) every day for two weeks. During the peak of egg laying, egg clusters were carefully harvested and transferred to petri dishes (2.5 cm diameter) lined with dampened filter paper and sealed by Parafilm. The hatchability was deduced from the following equation.

$$\mathrm{Hatching}\;\mathrm{rate}\;(\%)=\;\frac{The\;number\;of\;incubated\;eggs}{The\;total\;number\;of\;eggs}\;\times 100$$

#### 2.3.3. Measurements of Enzyme Activities and Vn Level

At 24 h, 48 h and 72 h post CNAP sublethal treatments via topical application, alive females were sacrificed in liquid nitrogen, homogenized in ice-cold sodium phosphate buffer (0.01 M, pH 7.2–7.4) (Beijing Solarbio Science & Technology Co., Ltd., Beijing, China) at ratio of 1:10 (*w*:*v*) using an electrically driven tissue homogenizer (TGrinder, Tiangen, Beijing, China). The cocktail was transferred into a 2 mL centrifuge tube and centrifuged at 10,000× *g* for 20 min at 4 °C, and supernatants separated from pellets were merged for measurements. SOD, CAT and peroxidase (POD) activity was assayed by commercial assay kits (Nanjing Jiancheng Bioengineering Institute, Nanjing, China) as per manufacturer’s instructions. An insect Vn enzyme-linked immunosorbent assay kit (Shanghai COIBO Biotechnology Co., Ltd., Shanghai, China) was used to quantify vitellin content from ovarian tissues (72 h), which was dissected in accordance with the protocols of Chen et al. [14]. Absorbance values at 405 nm (TAC), 240 nm (CAT), 470 nm (POD), 560 nm (SOD) and 450 nm (Vn) were measured using an Epoch2 multifunction microplate reader (BioTek Instruments, Highland Park, VT, USA). Each concentration at each time point comprised three biological parallels and three females per parallel (n = 3, N = 3).

### 2.4. Statistical Analysis

The LC values (LC_10_, LC_30_ and LC_50_), 95% confidence intervals and slopes were calculated based on probit analysis using PoloPlus (Version 1.0, LeOra Software, Berkeley, CA, USA). Statistics were implemented in Data Processing System [27]. Specifically, the Shapiro–Wilk test was used to test the normality of the distribution and Levene’s test for checking the homogeneity of the variance. Since the data of developmental indexes, fecundity-related proxies and enzymatic activities conformed to normal distribution, a one-way analysis of variance (ANOVA) followed by Tukey’s multi-comparison was performed to determine the significance among treatment means (see Supplementary Materials, Table S1). Data were presented as mean ± SEM (standard error of mean) of at least three independent biological replicates (N = 3), with differences considered statistically significant at *p* < 0.05. Prism version 9.0 (GraphPad Software, San Diego, CA, USA) was manipulated for visualization.

## 3. Results

### 3.1. Acute Toxicity and Safety Factor of CNAP

As detailed in Table 1, mortality data was linearly fitted with the toxic regression equation, with *p* values > 0.05. LC_50_ values of CNAP against eggs, 1st-instar larvae and NEAF were estimated as 90.11, 86.11 and 240.50 mg/L, respectively, with the strongest toxicity appeared at 1st-instar larval stage. Based on calculated safety factors, CNAP posed a moderate risk for embryos and 1st-instar larvae, while it was low-to-medium toxic to NEAF. The LC_10_ and LC_30_ data of divergent life stages were then applied to assess sublethal effects on physiological fitness of *H. axyridis*.

### 3.2. Sublethal Effects of CNAP on Developmental Traits

**Figure 1:**
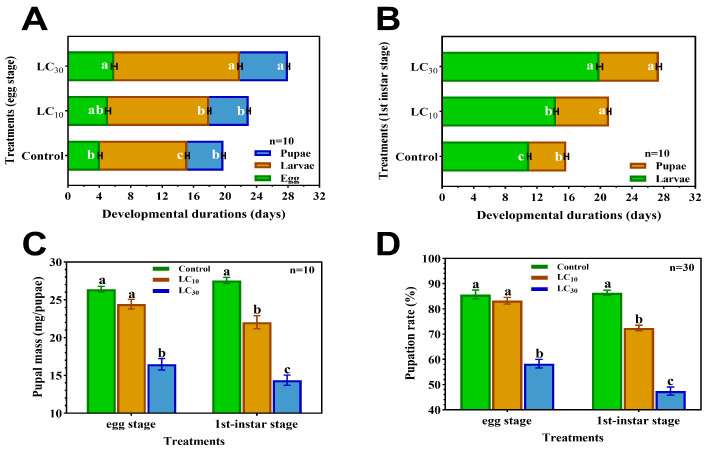
Toxic effects of CNAP exposure on development of *H. axyridis* at immature stages. (**A**) Embryonic, larval and pupal durations upon sublethal treatments at egg stage. (**B**) Durations of larvae and pupae grown from 1st instar subjected to sublethal treatments. (**C**) Fresh weight and (**D**) pupation rate of insects pupated from embryos and 1st instar treated by CNAP. Each histogram and error bar represented mean ± SEM. Different lowercase letters within the same panel denoted significant differences among groups (*p* < 0.05).

When eggs were treated with CNAP, although pupal mass (Figure 1A,C), pupation rate and embryonic and pupal stage developmental time of the LC_10_ group were not significantly different from those of the control, this exposure dose indeed caused significant retardation of the larval stage. Apparently, embryonic, larval and pupal durations were considerably prolonged by LC_30_ treatments, which also significantly mitigated pupal mass and pupation rate to 16.47 mg and 58.24% as compared to control (26.40 mg, 85.66%).

In terms of 1st-instar larvae exposed to CNAP (Figure 1B,D), both LC_10_ and LC_30_ significantly extended larval and pupal periods relative to control, mounting by 30.9% and 42.6% in the LC_10_ group, and 80.9% and 59.6% in the LC_30_ group, respectively. In addition, pupal weight and pupation rate were substantially decreased following LC_10_ and LC_30_ treatments, down by 20.1% and 16.1%, and 47.9% and 45.1%, respectively, in each group compared to control.

### 3.3. Sublethal Effects of CNAP on Female Egg-Laying Proxies

**Figure 2:**
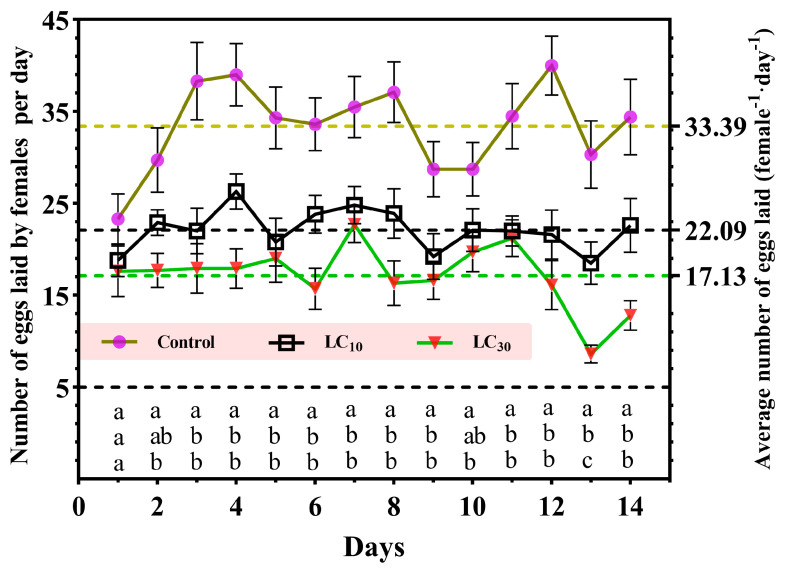
Effects of CNAP application on reproductive capacity of females within 14 days from the onset of oviposition. Scatter dots were expressed as mean ± SEM (n = 10). Different lowercase letters within each column denoted significant differences among groups on the same day (*p* < 0.05).

For each day in the course of observation, the number of eggs deposited was dramatically diminished by CNAP treatments versus control (Figure 2). Unlike the relatively stable egg laying illustrated in the LC_10_ group, albeit the considerable gap between the LC_30_ and control groups, curves representative of average fecundity displayed similar dynamics over time. From day 12 to 14, the largest recessions of 59.7–71.6% and 38.9–46.0% were overtly presented in the LC_10_ and LC_30_ groups compared to control, respectively. Average spawning within 14 days in the LC_10_ and LC_30_ groups was 22.09 and 17.13, respectively, far fewer than that of the control (33.39).

**Figure 3:**
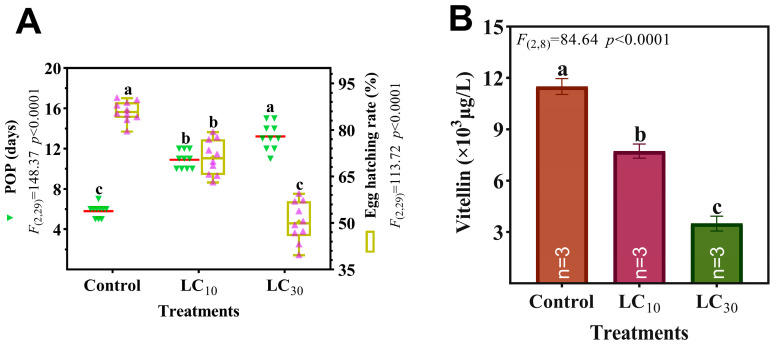
Toxic effects on POP and egg hatching rate (**A**) as well as vitellin level (72 h after exposure) (**B**) of females treated by CNAP. Red lines and green/purple triangles denoted means and specific values, while boundaries of boxes signified the 25/75th percentiles and whiskers indicated minimum/maximum, with horizontal lines as medians. Histograms plus error bars represented mean ± SEM. Different letters above marked significant differences among groups (*p* < 0.05).

**Figure 4:**
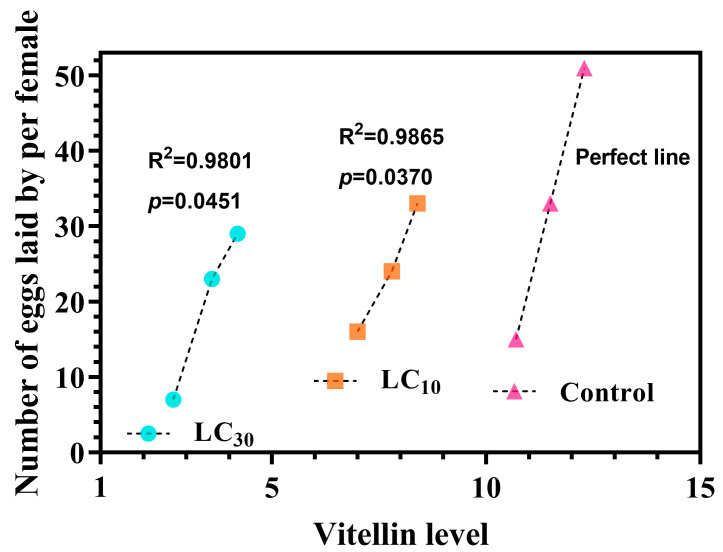
Pearson correlation analysis between vitellin level (72 h) and number of eggs laid by individual female at day 7 since oviposition. The minimum, median, and maximum values of each group (Figure 2, day 7) were aligned with the vitellin abundance (Figure 3B) accordingly.

In comparison to control, egg incubation rate was significantly mitigated by 17.3% and 41.4% (Figure 3A), while POP was substantially elongated by 7.4 days and 5.1 days upon LC_10_ and LC_30_ exposure to CNAP (Figure 3A), respectively. Figure 3B manifested a pronounced dose-dependent reduction of ovarian Vn level post CNAP sublethal treatments, down by 32.8% and 69.6% at LC_10_ and LC_30_, respectively. For both treated and control groups, a significant positive correlation existed between Vn level (72 h) and number of eggs laid per female (day 7 from the onset of oviposition) (Figure 4), suggesting Vn accumulation may directly affect spawning.

### 3.4. Sublethal Effects of CNAP on Female Antioxidant System

**Figure 5:**
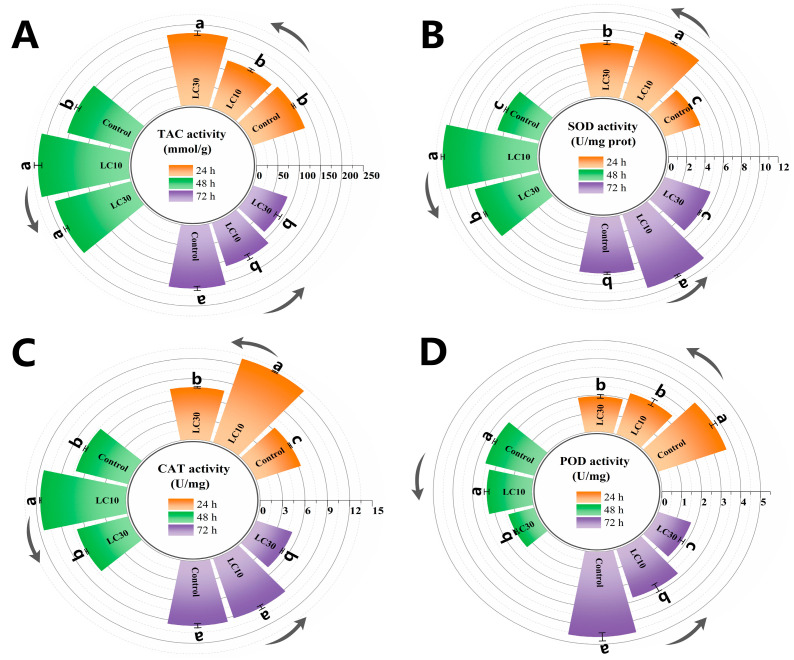
TAC (**A**), SOD (**B**), CAT (**C**) and POD (**D**) activity in females amenable to sublethal exposure of CNAP at 24, 48 and 72 h. Each histogram indicated the mean of three repetitions (n = 3) and error bars depicted SEM. Different lowercase letters denoted statistically significant differences among groups (*p* < 0.05).

The activities of four major antioxidant enzymes consistently soared in the control group with the passage of time, implying escalated oxidative defense with rising age. After sublethal treatments of CNAP, the antioxidant system was gravely compromised and even experienced a total collapse at 72 h under LC_30_ (Figure 5). Specifically, there was a significant diminution in POD activity relative to the control over time, down by 35.7–40.0% for LC_10_ and 47.0–61.8% for LC_30_ (Figure 5D). As compared to the control, TAC activity was firstly promoted by 37.2% in LC_10_ and 37.2–38.8% in LC_30_ group between 24 and 48 h (Figure 5A), which was then followed by a pronounced decrease of 27.9% (LC_10_) and 45.2% (LC_30_). This trend of stimulation ensuing inhibition also appeared for SOD and CAT activity in the LC_30_-treated group between 24 and 72 h (Figure 5B,C). It was intriguing to observe upsurges in SOD and CAT activity upon LC_10_ exposure throughout this bioassay, with peak levels of 10.31 (SOD, 48 h) and 12.97 (CAT, 24 h) versus control (4.75, 48 h; 6.33, 24 h) at the same time.

## 4. Discussion

Under realistic field conditions, insects are generally exposed to low lethal or sublethal concentrations of pesticides resulting from uneven spray and progressive degradation following initial applications [23]. It is therefore fundamental to deliberate over acute toxicity and sublethal effects of agrochemicals for sustainable conservation and harnesses of natural enemy insects.

Beneficial arthropods can encounter insecticides used against target pests either by direct contact, or by exposure to their residues on plant tissues, or through indirect consumption of contaminated prey [28,29]. Here we exposed *H. axyridis* to CNAP via the direct contact method, with the highest/lowest toxicity identified at the 1st-instar stage and NEAF, respectively. This finding was supported by the argument that the adult phase was often less susceptible than immature stages [12,30]. Similarly, the nymphal stage of *Orius insidiosus* (Heteroptera: Anthocoridae) harbored the least tolerance to sunflower stems grown from thiamethoxam-treated seeds [29]. The strongest toxicity against larvae relative to eggs and adults can be interpreted by embryo protection from egg chorion and advanced cuticular sclerotization in adults, which constitute mechanical resistance and hinder exogenous penetration of xenobiotics into integument. In addition, toxicity levels may be associated with detoxification enzyme activity and sensitivity of the action site across insect life cycles [16,30]. Due to more considerable glutathione s-transferase and acetylcholinesterase activity in *H. axyridis* adults than in larvae [31], it was tempting to speculate that lower enzymatic activities in 1st instar would not be able to detoxify and eliminate CNAP, thereby resulting in higher demise and sensitivity of this stage.

The compatibility of certain insecticidal compounds with beneficial organisms permits their incorporation into IPM systems [32]. Lines of evidence reveal that CNAP is ecofriendly for a number of beneficial insects and mites as well as mammalian, avian and aquatic organisms, rendering it a promising IPM candidate [7,33]. A previous report indicated that CNAP could effectively control insect pests while being compatible with *Chrysoperla carnea* (Neuroptera: Chrysopidae) [34]. Unlike this slight impact, intoxication risk classified by the safety factor was rated as moderate in this work, inferring ecological threats posed by CNAP for coccinellids. Nonetheless, Koch (2003) concluded that *H. axyridis* was not sensitive to some insecticides used for pest control. Specifically, spinosad, indoxacarb and pyriproxyfen harbored minimal toxicity against *H. axyridis*, but higher mortality (67–100%) was observed for carbaryl, chlorpyrifos fenpropathrin and cyhalothrin. Alternatively, CAP posed no acute toxicity risk to *Paederus fuscipes* (Staphylinidae: Coleoptera) but did repress *H. axyridis* populations at recommended field rates [13,22]. Chemical modes of action and interspecies variation may be responsible for differences in risk estimation.

Aside from mortality, pesticide exposure can also induce a suite of fitness alterations in beneficial arthropods [21], which basically involves detrimental effects on growth and development, survival and longevity, predatory or parasitic function, reproductive capacity, population demography, feeding and locomotion and enzymatic properties [23,24]. Ingesting artificial diets spiked with LC_30_-CNAP delayed larval development and significantly lessened pupation rate and pupal weight of *Helicoverpa assulta* (Lepidoptera: Noctuidae) [7]. Wang et al. [8] indicated that LC_10_ and LC_30_ CNAP significantly prolonged the nymphal development time of *Laodelphax striatellus* (Homoptera: Delphacidae) at 1st, 4th and 5th-instar stages following rice seedling dipping method. In addition to sublethal toxicity against target herbivores, CNAP treatments also go against fitness-related traits of their predators. As stated by Wu et al. [35], durations from 2nd instar to adult eclosion were retarded, while pupal weight of *Coccinella septempunctata* (Coleoptera, Coccinellidae) was mitigated via contact with LR_50_ dinotefuran and clothianidin. Using the same residual film method, Shan et al. [25] also demonstrated arrested development of larvae in *Chrysoperla sinica* (Neuroptera: Chrysopidae) exposed to LC_25_ emamectin benzoate and lambda-cyhalothrin. Meanwhile, LC_25_ indoxacarb, imidacloprid and lambda-cyhalothrin reduced pupal weight accompanied by an elongated pupal period. In this study, eggs and 1st-instar larvae were intoxicated by LC_10_/LC_30_ CNAP via the dipping method and topical application, respectively, with concurrent extension in embryonic, larval, and pupal durations as well as diminution in pupation rate and pupal weight. All these results suggest that despite variations in insect taxa, exposure routes and physicochemical properties of chemicals, sublethal doses of insecticides mostly stunt growth rate, extend developmental periods and deplete body mass of immature stages [21].

This phenomenon can be explained by antifeedant activity and energy imbalance resulting from toxicant exposure [32,36]. For example, *C. sinica* treated by four different insecticides at LC_25_ all strikingly attenuated predatory capacity of larvae [25]. CNAP exhibited prominent sublethal effects against nutritional profiles of *Agrotis ipsilon* (Lepidoptera: Noctuidae), manifested as diminished lipids, carbohydrates and proteins, thus impeding larval and pupal growth [33]. Though this was not explored in this study, we hypothesized that CNAP may limit predation, ingestion and digestion of *H. axyridis*, triggering nutrition deficiency and energy crunch that precluded the pupation of larvae. Alternatively, arrested durations and growth of preadults may be linked with the fact that more energy reserves are allocated for detoxification metabolism rather than for developmental purposes, as reported in *Ostrinia furnacalis* (Lepidoptera: Crambidae) subjected to CNAP [36]. It should be noted that compared to the egg stage, 1st instars treated by CNAP led to developmental parameters significantly different from those of the control group even at LC_10_, which further proved potent toxicity of CNAP to 1st-instar larvae.

Virtually all classes of insecticides interfere with insect reproduction through sublethal adverse effects on egg fertilization, oogenesis, ovulation, spermatogenesis and sperm motility [37]. For instance, LC_25_ indoxacarb, imidacloprid and lambda-cyhalothrin dramatically reduced the number and hatching rate of eggs oviposited by lacewing [25]. This study also found that LC_10_/LC_30_ CNAP significantly protracted POP and suppressed daily oviposition as well as the egg hatching rate. In line with these sublethal effects, Pan et al. [38] indicated similar alterations in fecundity, pre-oviposition period and egg hatchability in *Apolygus lucorum* (Hemiptera: Miridae) treated by cycloxaprid at LD_40_. Consistently in *H. assulta*, significant reproductive loss of females appeared after 3rd-instar larvae was administered with LC_30_ CNAP [7]. Since CNAP functions on insect RyRs to release calcium ions from cells and block muscle contraction [5], it may interfere with the sperm transfer/storage process and oviduct contractile activity in the female reproductive tract. Hence, elongation in POP may be attributed to disrupted copulation and lethargic egg laying. Such anomalous behaviors were echoed in *Podisus nigrispinus* (Heteroptera: Pentatomidae), *O. insidiosus*, *Frankliniella occidentalis* (Thysanoptera: Thripidae) and *P. fuscipes* [10,22,29,32], where extended POP and mitigated fecundity were observed post xenobiotic intoxication.

As the major egg storage protein, Vn generation, absorption and deposition is indicative of insect reproduction [14,39]. Among considerable nutritional resources, Vn played major nourishing roles pivotal for ovarian and embryonic development. Accordingly, the dose-dependent decrease in Vn content may cause a parallel decline in egg hatchability upon sublethal exposure to CNAP. Additionally, we pointed out positive correlations between Vn levels and number of deposited eggs. Likewise, Tufail and Takeda [39] confirmed that Vn accumulation was closely relevant to ovarian maturation and oogenesis. It was presumable that sublethal treatments of CNAP induced reproductive toxicity by regulating the synthesis and accumulation of Vn.

Most insecticides at sublethal concentrations do not kill insects but can disrupt endogenous enzymes to varying degrees [9]. Organismal cells are protected by SOD, CAT and POD that constitute a first-line antioxidant defense to counteract oxidative damage and scavenge free radicals, i.e., reactive oxygen species (ROS) [40]. T-AOC activity represents the overall status of the antioxidant system, including both enzymatic and non-enzymatic antioxidants [41]. We showed that redox balance was tremendously breached upon CNAP sublethal treatments, with SOD, CAT and TAC activity significantly elevated between 24 and 48 h; POD activity was constantly suppressed throughout the entire test period; all enzymatic activity descending at 72 h under LC_30_.

Coincided with our outcomes, antioxidant activities were also stimulated in *Pleonomus canaliculatus* (Coleoptera: Elateridae) and *Anomala corpulenta* (Coleoptera: Scarabaeidae), *C. sinica* subjected to CNAP [9] and tolfenpyrad [24], respectively, at sublethal doses, suggesting their protective roles against oxidative burst arising from these toxicants. By contrast, their activity during the whole developmental period of *C. sinica* was depleted by sublethal treatments with insecticides [25], in accordance with dropped POD activity and compromised systems herein, noticeably under L_30_ at 72 h. This inhibition can be ascribed to overproduction of ROS far exceeding the defensive capability, rendering failure of self-repair and the collapse of the antioxidant system. Intriguingly, SOD and CAT activity both peaked in the LC_10_ group between 24 and 72 h. This unexpected promotion positively caused by low doses of toxicants was also revealed for other insects [8,10], and may be interpreted as hormesis effects responsible for overcompensation of physiological homeostasis to exogenous perturbations.

When challenged with xenobiotic stressors, biological systems routinely fine-tune physiological homeostasis via tactics like energy reallocation and/or fitness trade-offs to sustain individual survivorship and development. In addition to elicited hormesis, enhanced TAC/SOD/CAT activity following CNAP sublethal exposure may be a compensatory mechanism derived from decreased oviposition, egg hatchability and Vn amount. Female adults gave priority to survival by shifting more energy to combat and detoxify CNAP instead of spawning. Still, genetic backgrounds underpinning this adaptive mechanism merit further dissection.

The present study only underlined the environmental hazard of CNAP to the harlequin ladybird since it serves as an efficacious biocontrol agent in natural realms. However, from a global perspective, *H. axyridis* rapidly disperses as an invasive alien species to Europe and many other areas of the world, menacing indigenous ladybird biodiversity and ecosystem services through resource competition and intra-guild predation [42]. Standing out for its polyphagy, broad-spectrum hosts and potent adaptivity to harsh conditions, populations of *H. axyridis* are extensively distributed in more than 59 countries outside Asia and raise concerns among the academic community [43]. Hence, opinions can be divided regrading whether to conserve or manage this arthropod, and answers may depend on native/colonized ranges where it occurs and ecological consequences that it engenders. Future investigations should notice the double-edged sword of *H. axyridis* introduction and evaluate the control efficacy of CNAP against this invasive predator in semi-field and field conditions. Moreover, the feeding behavior, functional responses, detoxification metabolism and underpinning molecular mechanisms of *H. axyridis* upon exposure to diamides will be deciphered in follow-up research.

## 5. Conclusions

In summary, the acute toxicity and developmental, reproductive as well as biochemical toxicity elicited by lethal and sublethal concentrations of CNAP to *H. axyridis* were investigated. The LC_50_ values of CNAP against *H. axyridis* across life stages were 86.11–240.50 mg/L, with the strongest toxicity to 1st-instar nymphs and risk level estimated to be medium, implying hazards for utilization of CNAP as an IPM component. Post CNAP sublethal exposure, we observed overall fitness depletion reflected by retarded immature stages and POP; dropped pupal weight and pupation rate; diminished fecundity, egg hatchability and vitellin accumulation; and dysfunctional antioxidant systems. All these proxies featured a concentration-dependent response over time in that LC_30_ (moderate lethal dose) induced more intense sublethal effects than LC_10_ (low lethal dose). This work may contribute to the development of improved IPM tactics that take compatibility of pesticides with nontarget arthropods into account.

## Figures and Tables

**Table 1:** Acute toxicity of CNAP to *H. axyridis* and its safety factor.

Life Stages ^a^	Slope ± SE ^b^	LC_50_ mg a.i.L^−1^ (95% CI ^c^)	LC_10_ mg a.i.L^−1^ (95% CI)	LC_30_ mg a.i.L^−1^ (95% CI)	χ^2^ (*df*) ^d^	*p* ^e^	TAP ^f^	Safety Factor
Embryos (50)	2.88 ± 0.35	90.11 (75.14–107.38)	32.31 (21.95–41.88)	59.22 (46.52–71.31)	4.74 (3)	0.1919	10 days	1.20–2.00 (medium)
1st-instar larvae (15)	1.34 ± 0.38	86.11 (44.14–154.15)	9.47 (0.50–23.81)	34.90 (8.23–61.69)	0.16 (3)	0.9837	24 h	1.14–1.91 (medium)
NEAF (60)	1.56 ± 0.20	240.50 (178.65–304.57)	36.09 (15.75–60.36)	110.68 (67.93–152.93)	0.6982 (3)	0.8736	24 h	3.21–5.34 (low to medium)

^a^ The replicates tallied with the number of individuals was listed in parentheses. ^b^ SE, Standard error of the slope. ^c^ CI, 95% confidence intervals. ^d^ Chi-square value (χ^2^) and degrees of freedom (df). ^e^ *p*-value derived from Chi square test > 0.05 indicated the log-logistic model provides acceptable description of data. ^f^ TAP, time after application.

## Data Availability

Experimental data are available from the corresponding author on request.

## Supplementary Materials

The following supporting information can be downloaded at: https://www.mdpi.com/article/10.3390/insects15100773/s1, Table S1: Summary of statics derived from one-way ANOVA analysis.

## Abbreviations

Total antioxidant capacity (TAC); superoxide (SOD); peroxidase (POD); catalase (CAT); lethal concentration that causes 10% and 30% mortality (LC_10_ and LC_30_); newly emerged adult females (NEAF); vitellin (Vn); pre-oviposition period (POP); median lethal concentrations (LC_50_); Cyantraniliprole (CNAP); integrated pest management strategies (IPM)
