# Supplementary material for: Exposure to Cyantraniliprole Adversely Impacts Fitness of Harmonia axyridis: Acute Toxicity and Sublethal Effects on Development, Fecundity and Antioxidant Responses
Source: Insects. 2024 Oct 6;15(10):773. doi: 10.3390/insects15100773 (PMC11508540; doi:10.3390/insects15100773)
Supplement: Supplementary file 1 [file insects-15-00773-s001.zip › insects-3186661-supplementary.pdf]

Supplementary Material

Exposure to cyantraniliprole adversely implicates fitness of *Harmonia axyridis*: acute toxicity and sublethal effects on development, fecundity as well as antioxidant responses

Tianshu Zhang☯[1,2], Yongda Yuan☯[1,2], Haiyuan Teng[1,2], Dongsheng Wang[1,2], Haotian Gu[*1,2]

[1]Shanghai Key Laboratory of Protected Horticultural Technology, Eco-Environmental Protection Research Institute, Shanghai Academy of Agricultural Sciences, Shanghai, 201403, China

[2] Shanghai Engineering Research Centre of Low-Carbon Agriculture (SERCLA), Shanghai, 201415, China

[*]**Corresponding author, to whom correspondence may be addressed:**

guhaotian@saas.sh.cn

☯ **Co-first authors. These authors contributed equally to this work.**

Table S1. Summary of statics derived from one-way ANOVA analysis.

**Contents**	***F***	**df (intergroup df, total df)**	***p***
Figure.1A egg	8.891	2,29	0.0011
Figure.1A larvae	120.196	2,29	0.0001
Figure.1A pupae	14.416	2,29	0.0001
Figure.1B larvae	233.73	2,29	0.0001
Figure.1B pupae	30.032	2,29	0.0001
Figure.1C egg stage	74.405	2,8	0.0001
Figure.1C 1st instar stage	96.925	2,8	0.0001
Figure.1D egg stage	87.609	2,8	0.0001
Figure.1D 1st instar stage	239.261	2,8	0.0001
Figure.2 day1	1.483	2,29	0.2449
Figure.2 day2	6.153	2,29	0.0063
Figure.2 day3	11.192	2,29	0.0003
Figure.2 day4	17.146	2,29	0.0001
Figure.2 day5	8.378	2,29	0.0015
Figure.2 day6	13.642	2,29	0.0001
Figure.2 day7	7.414	2,29	0.0027
Figure.2 day8	13.783	2,29	0.0001
Figure.2 day9	6.231	2,29	0.006
Figure.2 day10	3.497	2,29	0.0446
Figure.2 day11	8.672	2,29	0.0012
Figure.2 day12	19.216	2,29	0.0001
Figure.2 day13	17.973	2,29	0.0001
Figure.2 day14	12.471	2,29	0.0001
Figure.3A POP	148.37	2,29	0.0001
Figure.3A egg hatching rate	113.72	2,29	0.0001
Figure.3B	84.64	2,8	0.0001
Figure.5A 24h	56.942	2,8	0.0001
Figure.5A 48h	18.416	2,8	0.0027
Figure.5A 72h	29.649	2,8	0.0008
Figure.5B 24h	160.616	2,8	0.0001
Figure.5B 48h	352.975	2,8	0.0001
Figure.5B 72h	221.557	2,8	0.0001
Figure.5C 24h	1070.495	2,8	0.0001
Figure.5C 48h	302.885	2,8	0.0001
Figure.5C 72h	98.386	2,8	0.0001
Figure.5D 24h	33.662	2,8	0.0005
Figure.5D 48h	40.593	2,8	0.0003
Figure.5D 72h	55.72	2,8	0.0001
